# Virtual Reality as a Window into Sibling Aggression

**DOI:** 10.1007/s10802-025-01359-1

**Published:** 2025-09-06

**Authors:** Sheila R. van Berkel, Andrea L. Haccou, Catharina E. Bergwerff

**Affiliations:** Institute of Education and Child Studies, Leiden University, Leiden, The Netherlands

**Keywords:** Virtual reality assessment, Sibling aggression, Validity, Child and adolescent mental health, Sibling relationship

## Abstract

Sibling aggression is the most common form of domestic violence, which can have a negative impact on both child and adolescent mental health. The few previous studies that investigated aggression between siblings, assessed aggression primarily through self- or parent-report, with the limitation of reporter bias. The current study examined whether an interactive Virtual Reality (VR) experiment can provide a valid assessment of adolescents’ aggressive responses towards their sibling by testing congruence with other similar measures and by examining associations of known risk factors for sibling aggression with the aggression observed in the VR experiment. Pairs of young adolescent siblings (*N* = 26; aged 8–15 years) were invited to the lab to complete several questionnaires and participate in a custom-made interactive VR experiment. In the VR experiment, participants interacted with their virtual sibling both verbally and physically. Participants’ responses to the virtual sibling’s behavior, designed to provoke anger and aggression, were observed during two different VR scenarios. Results showed that observed aggression as measured in the VR experiment was related to self-reported aggression, but not to parent- or sibling-reported aggression. Individual factors (e.g., behavioral problems) or sibling factors (e.g., age difference between siblings) were not related to observed aggression. Of the investigated family factors, only the perceived quality of the father-child relationship was associated with observed aggression. Despite the limited alignment with parent- and sibling-reported aggression, these findings highlight the potential of VR-based assessments to complement self-report methods, emphasizing the need for a multimethod approach to capture the complexities of sibling aggression.

Sibling aggression is the most common form of domestic violence and has been associated with child and adolescent mental health problems and delinquency (Van Berkel et al., 2018). Sibling aggression is often not recognized as being more harmful than normative sibling conflict (Perkins & Grossman, 2020). However, sibling aggression refers to clinically significant levels of violence, resulting in physical, psychological or social harm (Ingram et al., 2020). Moreover, research suggests that aggression among siblings is related to aggression in other contexts, that it is stable over time (Fite et al., 2021), and increases the risk of delinquency both for siblings subjected to as siblings exhibiting the aggression (Brett et al., 2023; Van Berkel et al., 2018). Although research on sibling aggression is expanding, there is debate about the best methods for valid assessment. The large majority of studies on sibling aggression uses self-report measures (Brett et al., 2023; Fite et al., 2021; Ingram et al., 2020; Van Berkel et al., 2018), which may have limited ecological validity due to social desirability, memory bias, and possible problems with correctly identifying incidents due to a relatively high tolerance for aggression in sibling relationships (Tucker et al., 2025). The present study investigates the validity of a newly developed interactive Virtual Reality (VR) experiment for assessing sibling aggression.

One of the main challenges in the field of studying aggression, is the difficulty of capturing aggression in an ecologically valid manner. The handful of studies that investigated aggression between siblings thus far, assessed aggression primarily through self- or parent-report, with the limitations of reporter bias and social desirability (Kirsch et al., 2024; Lawrence et al., 2023). Studies assessing aggression in other contexts (including aggression in peer groups or at school) often use questionnaires, vignettes or computer experiments which may induce bias due to a lack of immersion, adaptation, and ecological validity (Verhoef et al., 2022). Similar concerns have been raised regarding using self-report and observations to assess aggression. For instance, ethnic differences seem to impact self-report and cultural biases may impact observation (Lampe et al., 2023). Further, given informant discrepancies on measures of aggression in children (Perry et al., 2021), a multimethod approach may be warranted. Implementing an interactive VR experiment could overcome several of these issues and shows promise for validly assessing aggression in youth (Verhoef et al., 2022).

First, compared to questionnaires or vignettes, a VR experiment may enhance the immersion (sense of being there) of adolescents in social situations with their (virtual) sibling, potentially evoking aggressive responses. This approach appears to be more ecologically valid for assessing aggression, as observing adolescents’ primary responses in provocative situations may prevent them from giving socially desired answers in questionnaires or vignettes. This seems to be corroborated by a recent study that used VR to assess aggression in children, which found that their VR experiment was more sensitive to capture individual differences in aggressive responding compared to vignettes (Verhoef et al., 2022). Furthermore, supporting convergent validity, they found small to large significant correlations between anger, hostile intent attribution, revenge goals, and aggressive responding measured in a VR experiment and with vignettes. Second, interactive VR provides meticulous experimental control, facilitating standardized scenarios across participants while being able to fine-tune the scenario to the responses of the participant and to adhering ethical protocols by regulating social events within the VR environment (Verhoef et al., 2022). Using VR may therefore be the way to move forward in assessing sibling aggression, next to questionnaires and/or vignettes. In the current study, we investigated whether aggression measured in a newly developed VR experiment was related to aggression measured through self-report, parent-report, and sibling-report to gain insight into the convergent validity.

In addition, we investigated construct validity by examining associations between sibling aggression measured in the VR experiment with child and family factors which have been associated with sibling aggression in previous studies (Brett et al., 2023; Tippett & Wolke, 2015). Sibling aggression is embedded in the family system, and previous studies have shown that family factors are related to the development of sibling aggression. For instance, it is suggested that high levels of conflict between parents (including interparental violence), parental differential treatment, and a hostile or abusive parent-child relationship may be risk factors for the development of sibling aggression and abuse (Ingram et al., 2020; Loeser et al., 2016; Portner & Riggs, 2016; Van Berkel et al., 2018; Wolke & Lereya, 2015). On the other hand, family factors may function as protecting factors. For instance, it has been found that positive parent-child relationships may have a positive effect on sibling relationships (Portner & Riggs, 2016).

Other studies suggest that individual risk factors are associated with the development of sibling aggression. For instance, forms of internalizing behavior problems (such as depressive symptoms) and externalizing behavior problems (including substance abuse) seem to be related to sibling aggression (Lawrence et al., 2023). The link between internalizing problems and sibling aggression is further supported by theories on how anxiety influences aggression during childhood (Granic, 2014). In addition, gender and sibling gender combination has been associated to sibling aggression. However, results on gender and sibling factors are somewhat inconsistent (Kirsch et al., 2024). For instance, even though previous studies found that same-gender sibling dyads showed increased conflict compared to mixed-gender sibling dyads (Hehman et al., 2023), a recent study did not find a difference between brothers and sisters for sibling aggression (Kirsch et al., 2024). Despite the inconsistent findings, these factors might still shed light on the construct validity and antecedents of sibling aggression.

The aim of the current study is to investigate the utility of an interactive VR experiment as a means of assessing adolescents’ aggressive tendencies within the dynamics of sibling relationships. As this is a newly developed experiment, we conducted a first-phase pilot, in which we assessed the convergent validity. We expected that the level of aggression between siblings as measured by the VR experiment would be related to (a) self-reported aggressive responses to the sibling in hypothetical situations using vignettes, (b) parent report of child aggression using a questionnaire, (c) sibling report of aggression of the adolescent towards this sibling using a questionnaire, and (d) self-reported aggression between siblings using a questionnaire. A secondary goal of this study was to examine whether sibling aggression as measured in a VR experiment is related to individual and sibling factors (age difference between siblings, gender composition, and behavioral problems), and to family factors (parental conflict, differential treatment, and parent-child relationship). This was investigated to provide insight in both the antecedents of sibling aggression and the construct validity of the VR experiment.

## Method

### Sample

Young adolescents between the ages of 8 and 15 years were recruited from four schools, a (non-clinical) youth association, and an out-of-school care setting in nearby cities of the Leiden University in the Netherlands. The adolescents were provided with a presentation of the study, during which they could ask questions about participation. In addition, they received a flyer containing a QR-code, which they could scan to register online for participation when their sibling and one of their parents wanted to participate as well. Furthermore, parents were informed about the study through the digital newsletter of the respective school or organization, including a link for online registration. Adolescents were eligible to participate with their sibling, if their sibling (a) was aged between 8 and 15 years as well, (b) was biologically related to them (full or half), and (c) primarily lived in the same household. Due to concerns about homogeneity, as previous studies have suggested that sibling relationship quality may differ between siblings based on duration of co-residence, age difference, and genetic relatedness (Perry & Price, 2017; Sanner et al., 2018; Tanskanen & Danielsbacka, 2021) stepsiblings, adoptive siblings, and foster siblings as well as twin siblings were excluded from participation. Participants who could not understand the Dutch language were excluded, as all questionnaires had to be filled out in Dutch. Adolescents with severe psychological problems were excluded, as we aimed to include a general population sample. In addition, parents were asked whether there were any conditions that could impede participation in the VR experiment such as adolescents having photosensitive epilepsy, or serious visual or auditory impairments (Alsem et al., 2023). If parents indicated any impeding condition adolescents would be excluded. Prior to participation, informed consent was obtained from the participating parents and participating siblings aged 12 years or older. Participating siblings younger than 12 years old provided informed assent.

Twenty-six adolescents and one of their siblings were included in the current study, resulting in a total sample of 52 adolescents. This sample size was considered adequate to test both our hypotheses, as indicated by the a priori power analyses conducted in G*power 3.1.9.7 (Faul et al., 2009). The minimum required sample size for a linear regression: random model, with power 0.80 and α = 0.05 for detecting a medium effect was *N* = 42 for a model with three predictors and *N* = 49 for a model with five predictors. The older siblings of the participating sibling dyads were between 10.9 and 15.6 years old (*M* = 13.2, *SD* = 1.1) and the younger siblings were between 8.6 and 14.4 years old (*M* = 10.9, *SD* = 1.2). Of the older siblings 58% (*N* = 15) identified as male and the remaining 42% (*N* = 11) identified as female. Of the younger siblings 42% (*N* = 11) identified as male and the remaining 58% (*N* = 15) as female. The distribution of sibling gender configuration of the participating dyads was as follows: 5 boy-boy (19%), 5 girl-girl (19%), 10 older boy-younger girl (39%), and 6 older girl- younger boy (23%). All participants were biological (full) siblings and the majority lived with both biological parents (81%). Parents of the remaining 19% were divorced and the adolescents lived with each parent alternately. All participating parents were the biological parent of the adolescents and the majority identified as female (85%, 12% identified as male and one parent identified as having another gender). All adolescents regarded one of their parents as their father and one of their parents as their mother. Most of the sibling dyads had no other siblings, but 27% had one to three additional siblings. The sample was of a high educational level, with 54% of the sibling dyads having one parent with a high educational level (academic or higher vocational schooling) and 35% of the sibling dyads having both parents with a high educational level. All adolescents were born in the Netherlands and 23% of the sibling dyads had at least one parent that was born outside the Netherlands.

### Procedure

The sibling dyads and one of their parents were invited to the university lab. Both siblings of each dyad individually participated in a custom-made interactive Virtual Reality (VR) experiment designed to assess aggression. In addition, they individually completed several digital questionnaires and answered some questions in response to video vignettes. Participants were filmed when conducting the VR experiment and the video vignettes, so that their verbal and behavioral responses could be coded afterwards. The order of the tasks for the older and the younger sibling was counterbalanced between families. Furthermore, the parent completed several digital questionnaires on family demographics and child behavioral problems. During the lab sessions the three family members had no contact and completed the tasks in separate rooms. After finishing the data-collection, 15 gift vouchers worth 50 euros were raffled among the participating sibling dyads (i.e., a voucher of 25 euro per sibling). The study was pre-registered (AsPredicted# 90956) and ethical approval was provided by the Research Ethics Committee of the Institute of Education and Child Studies of Leiden University (registration number ECPW2021/333).

#### Virtual Reality Paradigm

The interactive VR experiment was developed based upon licensed software of a VR company (CleVR) in combination with a newly developed paradigm (adapted from Alsem et al., 2023). The VR experiment consisted of three immersive environments in which participants could interact with their virtual sibling, both verbally and physically. Participants wore a head-mounted display, a noise-cancelling headphone and held a controller in each hand. They could walk around freely (within a 3 m^2^ area), and they could move and manipulate objects in the virtual environment with their controllers. Participants were told that, unlike video games, interaction in the VR experiment followed the same rules as everyday life. They choose an avatar that resembled their sibling in the virtual environment and were made to believe that this avatar was controlled by their sibling from another room in the lab. In reality, a trained research assistant controlled the avatar.

The VR experiment consisted of three scenarios which were presented to the participants in fixed order. Before starting each scenario, participants entered a waiting room (envisioned as a small garden of a family house) in which they could get familiarized with the virtual environment and where they received the first instructions for the game they were about to play with their sibling when entering the scenario. In the first scenario participants sat on a couch in a living room with their virtual sibling and were told that they could watch television programs together. This first scenario was a neutral setting in which the participants could practice with the controllers. In the two other scenarios participants played a competitive game against their virtual sibling on a playground: (1) building the highest tower of blocks within 30 s and (2) hit all cans from a table within as few throws as possible, with a maximum of five throws. Participants were told that the winner of the game would get 5 euros. The research assistant controlled the games in such a way that it was impossible for the participant to win. Furthermore, the virtual sibling showed behavior that could provoke anger and aggression in the participant, by frustrating the game of the participants (knocking down the tower of blocks) or teasing the participant (for example by stating that the participant was not performing well and would definitely lose the game). The first provocations were provided to all participants in the same manner, to facilitate comparison between individuals. After these scripted provocations, the research assistant could adjust the responses of the virtual sibling to the participant’s behavior. The duration of these scenarios was on average 0:56 min (*SD* = 0:22 min, range = 0:25 ~ 3:07 min). Participants’ verbal and behavioral responses in these two scenarios were coded (see measures).

Directly after the VR experiment, participants were asked if they liked interacting with their sibling in the VR environment. Further, they were asked if they found the VR experiment realistic, whether they had noticed anything strange, and whether their sibling acted and responded the way they would normally do. The responses on these questions were used as indicators of whether participants believed that they were indeed interacting with their sibling in de VR experiment and thus whether the manipulation was successful. Based on their answers two coders indicated whether the manipulation was successful (yes or no). After answering these questions, participants were debriefed. All participants enjoyed the VR experiment and none of the participants showed any signs of anger towards their sibling or towards the research assistant after the debriefing.

### Measures

#### Observed Aggression in VR

Video recordings of the participants’ verbalizations and behaviors during the two competition scenarios of the VR experiment were double coded by two trained coders. The real-life behaviors of the adolescents were coded irrespective of whether the behavior was visible in the virtual reality. For example, some participants stuck out their tongue to their sibling or tried to kick down the tower of their sibling, although this is virtually not possible and the act was not visible in the VR the behavior was coded as aggression. To measure aggression, a coding system based on Kempes et al. (2008) was used. In line with previous studies (Kempes et al., 2008; Verhoef et al., 2022), aggression was coded as sibling-directed physical aggression (e.g. throwing an object to the virtual sibling, hitting or kicking the virtual sibling), psychological aggression (e.g. sticking out tongue to virtual sibling, scolding, insulting or bullying virtual sibling), or task-directed aggression (sabotaging the game of the virtual sibling e.g. by destroying the virtual sibling’s tower or by stealing the virtual sibling’s balls or blocks). This resulted in three measures of aggression reflecting the total frequencies of observed physical, psychological, and task-directed aggression in the two VR scenarios. Interobserver reliability was moderate for sibling-directed aggression with intraclass correlations (single rater, absolute agreement) for physical aggression of 0.52 and for psychological aggression 0.53. The moderate interobserver reliability was likely due to the small number of video fragments and the limited number of observations of physical and verbal aggression, which caused small deviations between coders to considerably affect the reliability. For task-directed aggression interobserver reliability was good with an intraclass correlation (single rater, absolute agreement) of 0.82. To improve coding, all scenarios in which there was disagreement between the two coders were re-coded by the first author, who is an experienced coder of sibling interactions.

#### Vignette-based Aggressive Response in Social Situations

Aggressive responses in social situations with the participating sibling were measured with three video vignettes of hypothetical social problems of the Social Information Processing Test (SIPT; Van Rest et al., 2020). Adolescents were instructed to watch each video while imagining that they and their participating sibling were two of the actors in the specific situation. After viewing each vignette on a laptop, adolescents were asked how they would respond if the event would occur between them and their sibling. Research assistants coded the responses of the adolescents as being assertive, aggressive or passive (Van Rest et al., 2020). The task was filmed and the responses of the adolescents were double coded by the first and second author. Inter-rater reliability kappa’s for the three videos were moderate (*κ* = 0.50), to substantial (*κ* = 0.78 *κ* = 0.80). The moderate kappa for one of the video scenarios was due to a misinterpretation of the coding system by one of the research assistants, which resulted in a systematic deviation between her coding and that of the other raters. Interrater reliability without these specific codes resulted in an adequate kappa of 0.72. The first author checked all dissimilarities between the two ratings, by watching the videos a second time and deciding on which code to use. To compute a total score for aggressive responses towards the participating sibling the number of aggressive responses was counted with a possible range of 0 to 3.

#### Parent-reported Aggression

The 18-item aggression subscale of the Child Behavior Checklist (CBCL 6–18) was used to measure parent-reported aggression (Achenbach, 2009; Verhulst et al., 1996). The participating parents indicated for both participating children individually whether they observed any of the described behaviors in the last six months on a three-point scale (0–2). A total score was calculated using a sum score with a possible range from 0 to 36. The internal consistency was good for both the older sibling *α* = 0.89 and the younger sibling *α* = 0.86.

#### Self-reported and Sibling-reported Aggression between Siblings

Aggression between siblings was measured with the subscales minor physical assault (5 items) and psychological aggression (7 items) of the sibling version of the Conflict Tactics Scale (CTS-SP; Carvalho Relva et al., 2013). Adolescents filled out this questionnaire twice. First, they indicated on a 7-point Likert scale ranging from never (1) to more than ten times in the past year (7), whether they showed specific behaviors during conflicts with their participating sibling (self-report). Subsequently the adolescents indicated whether their participating sibling showed these specific behaviors during mutual conflicts (sibling-report). Internal consistencies ranged from *α* = 0.73 to *α* = 0.87 for physical assault and from *α* = 0.71 to *α* = 0.91 for psychological aggression. For each sibling separate scores were computed for physical and psychological aggression and for self-report and sibling-report by calculating mean scores with a possible range of 1 to 7.

#### Individual and Sibling Factors

##### Behavioral Problems

Externalizing and internalizing behavioral problems were measured with the Child Behavioral Checklist (CBCL 6–18; Achenbach, 2009; Verhulst et al., 1996). A sum score was computed on all 35 items of two subscales on externalizing behavior (i.e., rule-breaking behavior, aggressive behavior; possible range 0–70). Similarly, a sum score was computed on the 32 items of the three subscales concerning internalizing behaviors (i.e., anxious/depressed, withdrawn/depressed, and somatic complains; possible range 0–64). Internal consistencies were adequate for both the older sibling *α*_externalizing_ = 0.87, *α*_internalizing_ = 0.75 and the younger sibling *α*_externalizing_ = 0.88, *α*_internalizing_ = 0.78.

##### Characteristics Sibling Dyad

To examine whether characteristics of the sibling dyad were related to observed aggression in the VR experiment, the age difference between the participating siblings was computed in months. Sibling gender combination was created as a binary measure with a category for same gender siblings and for different gender siblings, as comparing all four groups of sibling gender combinations was not possible due to the small sample size.

#### Family Factors

##### Interparental Violence

The subscales verbal aggression (6 items) and minor physical assault (3 items) of the Conflict Tactics Scale (CTS; Straus et al., 1996) were used to measure interparental violence in two-parent families. Parents indicated whether they or their partner showed the described behaviors and how often they showed this behavior within the past 12 months on a 7-point Likert-scale (1 = this never happened, 2 = not in the past year, but it happened before, 3 = once in the past year to 7 = happened more than ten times in the past year). Parents who indicated that they currently had no partner did not fill out the CTS (*N* = 5). For almost all parents who indicated they had a partner, this partner was the other biological parent of the children, only in one family the partner was a stepparent. A mean score was computed (possible range 1–7) with higher scores indicating more aggression between parents. Internal consistency was adequate *α* =. 75.

##### Parent-Child Relationship Quality

All participating adolescents completed the child version of the Parent-Child Interaction Questionnaire Revised (PACHIQ-R) twice (all participants indicated to report on their father and their mother) as a measure of parent-child relationship quality (Lange et al., 2002). Adolescents indicated on a 5-point Likert scale (ranging from 1 = never to 5 = always) to what extent the statements were applicable. Mean scores for fathers and mothers were computed of all 25 items concerning perceived parental acceptance and conflict resolution. Internal consistency was good for the older sibling *α*_mother_ = 0.85, *α*_father_ = 0.90 and ranged from adequate to good for the younger sibling *α*_mother_ = 0.79, *α*_father_ = 0.89.

##### Perceived Differential Parenting

To measure differential treatment of siblings by their parents, the five items of the subscale measuring affection of the Sibling Inventory of Differential Experience (SIDE; Daniels & Plomin, 1985) was filled out by both siblings on their father and their mother. To gain an indication of the extent of differential affection, rather than the extent to which one child was favored over the other, the absolute deviation from equal treatment was computed (see Feinberg et al., 2003). Therefore, the answers on the 5-point Likert scale (1 = much better for my sibling than for me, 2 = a bit better for my sibling, 3 = similar for the both of us, 4 = a bit better forQuery ID="Q5" Text="The citation(s) (Feinberg et al., 2003) is present in the text, but reference is missing in the reference section. Could you please provide the reference or delete the text citation." Resolved="yes" me, 5 = much better for me than for my sibling) were recoded with 0 = equal treatment (original score 3), 1 = a bit different (original scores 2 and 4), and 2 = clearly different (original scores 1 and 5). Mean scores, with a possible range from 0 to 2, for fathers and mothers separately were computed for each sibling. The internal consistency was adequate for the older sibling concerning father *α*_father_ = 0.79 and questionable for the items concerning mother *α*_mother_ = 0.61. For the younger sibling, the internal consistency was good concerning both parents *α*_mother_ = 0.77, *α*_father_ = 0.87.

### Analyses

All variables were inspected for possible outliers, defined as values outside a 3.29*SD range around the mean (Tabachnick et al., 2013). All three measures of observed aggression in the VR experiment, parent-reported aggression, internalizing behavior and both maternal as paternal differential affect contained one or two outliers on the high end of the scales. These outliers were winsorized to fall within the accepted range, aligning them with a normal distribution (Tabachnick et al., 2013). After winsorizing these outliers these variables were positively skewed (with skewness ranging from 1.38 to 2.41) as can be expected in a general population sample. All other measures were normally distributed and none of the variables contained missing values.

To investigate the validity of the interactive VR experiment in assessing aggression between siblings, associations between vignette-based aggressive responses in social situations, aggression between siblings (self-report and sibling-report), parent-reported aggressive behavior and the three measures of observed aggression in the VR experiment were computed. Subsequently these associations were tested while controlling for three covariates in separate analyses: age and gender of the participant, and whether the manipulation of the VR experiment was successful. To test associations between sibling aggression and individual, sibling, and family factors with a concise number of analyses, a sum score of the three measures of observed aggression was computed and two additional models were analyzed: one including individual and sibling factors and one including family factors.

To account for the nesting of siblings within families, we investigated the influence of the nesting on the outcome measures by estimating two-level multilevel models, using the Linear Mixed-effects model in SPSS 29.0. Intra-class correlation of the unconditional model (i.e., the model without predictors) was 0.24 for physical aggression, 0.14 for psychological aggression and 0.05 for object-directed aggression, indicating that 5–24% of the variance in observed aggression was explained by within-family variance between siblings. Given that a considerable portion of the variance was explained by the nesting of siblings within families, multilevel analyses with a random intercept were conducted to test the hypotheses. All variables were standardized before adding them to the multilevel analysis, so estimates could be interpreted as betas. Maximum likelihood was used when estimating the multilevel models to compare differences in −2 log likelihood (−2ΔLL) as an indicator for improved model fit.

## Results

### Descriptive Statistics

Descriptive statistics of all measures of aggression, the three covariates (gender, age, and successfulness manipulation VR), the variables concerning family and individual factors are presented in Table 1. Most participants showed some form of aggression during one of the VR scenario’s (65%, *N =* 34). This percentage is comparable to the 62% (*N =* 32) of participants that indicated to respond with aggression in one of the vignettes of the SIPT. Moreover, all three forms of aggression that were observed during the VR experiment had, despite the low means, a considerable range with physical aggression ranging from 0 to 8 events (after winsorizing: *M =* 0.27; *SD* = 0.60), psychological aggression ranging from 0 to 13 events (after winsorizing: *M* = 0.86; *SD* = 1.78), and task-directed aggression ranging from 0 to 15 events (after winsorizing: *M* = 1.73; *SD* = 2.01).

**Table 1:** Descriptive statistics of older and younger siblings

	Total (*N* = 52)	Older sibling (*N* = 26)	Younger sibling (*N* = 26)
	*M* (*SD*)	*M* (*SD*)	*M* (*SD*)
Age	12.0 (1.64)	13.21 (1.06)	10.86 (1.23)
Age difference (in months)	2.35 (0.76)	NA	NA
Gender (% males (*n*))	50% (26)	58% (15)	42% (11)
Same gender sibling dyad (% (*n*))	39% (20)	NA	NA
Parent-reported aggression	3.60 (4.1)	3.69 (4.48)	3.54 (3.92)
Aggression in social situations (vignette)	0.81 (0.77)	1.04 (0.87)	0.58 (0.58)
Physical aggression towards sibling (Self)	2.56 (1.04)	2.45 (0.92)	2.67 (1.16)
Psychological aggression towards sibling (Self)	3.27 (1.15)	3.41 (1.52)	3.14 (1.18)
Physical aggression towards sibling (Sibling)	2.92 (1.52)	2.62 (1.40)	3.22 (1.60)
Psychological aggression towards sibling (Sibling)	3.52 (1.54)	3.16 (1.52)	3.87 (1.49)
Observed physical aggression (VR)	0.27 (0.60)	0.27 (0.60)	0.27 (0.60)
Observed psychological aggression (VR)	0.86 (1.78)	1.10 (2.17)	0.62 (1.27)
Observed task-directed aggression (VR)	1.73 (2.01)	1.73 (2.03)	1.73 (2.03)
Successful manipulation VR experiment (% (*n*))	71% (37)	58% (15)	85% (22)
Child internalizing problems (parent report)	4.73 (3.87)	5.04 (3.68)	4.42 (4.10)
Child externalizing problems (parent report)	5.02 (5.42)	5.12 (5.54)	4.92 (5.41)
Inter-parental violence (parent report, *n* = 21)	1.67 (0.64)	NA	NA
Parent-child relationship quality			
Father	4.03 (0.43)	3.97 (0.46)	4.08 (0.41)
Mother	4.02 (0.37)	3.99 (0.41)	4.05 (0.34)
Perceived differential affect			
Father	0.16 (0.21)	0.17 (0.21)	0.15 (0.22)
Mother	0.15 (0.21)	0.13 (0.20)	0.17 (0.23)

NA = not applicable, ‘Self’ refers to self-report, ‘Sibling’ refers to sibling report, VR refers to the Virtual Reality experiment

Both physical and psychological aggression towards a sibling were reported frequently by both older and younger siblings regarding their own as well as their siblings’ behavior, with 73% (*N* = 19) of the older siblings and 81% (*N* = 21) of the younger siblings indicating to have used physical aggression towards their sibling at least once in the past year. For psychological aggression these percentages were 89% (*N* = 23) for the older siblings and 92% (*N* = 24) for the younger siblings. Parent report indicated relatively low levels of child aggression, child problem behavior, and interparental violence, as can be expected in a general population sample (Table 1). Adolescents were on average positive regarding their relationship with their parents and reported only minimal differential parenting (Table 1).

### Bivariate associations between Reported and Observed Aggression

To assess the convergent validity of the VR experiment, associations between vignette-based, parent-reported, sibling-reported, and self-reported aggression and each of the three measures of observed aggression in the VR experiment were tested. This resulted in six separate multilevel models of which the results are presented in Table 2.

**Table 2:** Fixed effects of multilevel models for convergent validity of observed aggression in the VR experiment

	Physical Aggression		Psychological Aggression		Task-directed Aggression	
	β	−2ΔLL	β	−2ΔLL	β	−2ΔLL
Parent-reported aggression	0.08	0.31	0.13	0.81	0.05	0.71
Aggression in social situations (vignette)	0.05	0.12	0.09	0.45	0.21	2.39
Physical aggression towards sibling (self)	0.29^*^	4.53^*^	− 0.04	0.07	0.04	0.09
Psychological aggression towards sibling (self)	0.32^*^	5.37^*^	0.18	1.63	0.27^*^	3.94^*^
Physical aggression towards sibling (sibling)	0.19	1.44	− 0.05	0.10	0.16	1.33
Psychological aggression towards sibling (sibling)	0.07	0.24	− 0.07	0.24	.19^a^	1.78^a^

* p <.05; ^a^ this model did not achieve convergence, indicating a poor model fit and possibly poor validity of the results. ‘Self’ refers to self-report, ‘Sibling’ refers to sibling report, −2ΔLL refers to the difference in −2 log likelihood of the model presented and the unconditional model

First, vignette-based aggressive responses were not related to VR assessed aggression. Second, none of the VR measures of aggression were significantly related to parent-reported aggression or sibling-reported aggression (see Table 2). Notably, the model for observed task-directed aggression as measured by the VR experiment predicted by sibling-reported psychological aggression towards the sibling did not achieve convergence, indicating that the model poorly fitted the data and that the result may not be valid. The result of this multilevel model was, however, similar to results of Pearson correlation analyses between task-directed aggression and sibling-reported psychological aggression, *r* (51) = 0.19, *p* =.19. Therefore, we chose, despite its problem with achieving convergence, to include this model in Table 2.

Finally, self-reported aggression was related to aggression as measured by the VR experiment. Observed physical aggression was positively related to both types of self-reported aggression towards the sibling. In addition, observed task-directed aggression was positively related to self-reported psychological aggression towards the sibling.

### Associations between Reported and Observed Aggression including Covariates

To assess the stability of the associations between reported aggression and observed aggression in the VR experiment, all six multilevel models for the three measures of observed aggression were repeated, while controlling for one of the three covariates. These analyses showed similar results as the bivariate multilevel analyses with significant associations for observed physical aggression with both physical (β_controlled for age_ = 0.29, *p* =.037; β_controlled for gender_ = 0.29, *p* =.039; β_controlled for successfulness manipulation VR_ = 0.30, *p* =.031) and psychological (β_controlled for age_ = 0.34, *p* =.017; β_controlled for gender_ = 0.31, *p* =.025; β_controlled for successfulness manipulation VR_ = 0.42, *p* =.004) self-reported aggression towards the sibling. In addition, observed task-directed aggression remained positively related to self-reported psychological aggression towards the sibling when controlling for age (β = 0.27, *p* =.049) and successfulness of the manipulation (β = 0.33, *p* =.025) but was no longer significant when controlling for gender (β = 0.27, *p* =.054). Overall, these multivariate analyses showed that the results were rather stable.

### Associations between Individual, Sibling, and Family Factors and Observed Aggression

Finally, associations between individual, sibling, and family factors and observed aggression in the VR experiment were analyzed. To limit the number of analyses the three measures of observed aggression were summed into one measure of total observed aggression in the VR experiment. To prevent overfitting the analysis or running an underpowered analysis, we decided to not include all predictors into one model. Instead, the associations with individual, sibling, and family factors were tested in two multilevel models (Table 3). One model testing the most proximal factors directly related to the siblings involved and the second model testing the more distal family factors. The model testing associations with individual and sibling characteristics showed no significant association between these factors and observed aggression in the VR experiment.

**Table 3:** Fixed effects of multilevel models for associations of Individual, sibling and family factors with observed aggression in the VR experiment

		Total Observed Aggression VR experiment	
	−2ΔLL	β	*p*
Model 1: Individual & Sibling factors (*n* = 52)	3.01		
Age difference between siblings		0.15	0.330
Same gender siblings		− 0.00	0.982
Older or younger sibling		− 0.06	0.671
Adolescent externalizing behavior (parent report)		0.16	0.334
Adolescent internalizing behavior (parent report)		0.11	0.477
Model 2: Family factors (*n* = 42)	9.60		
Inter-parental violence (parent report)		− 0.28	0.089
Parent-child relationship quality- Father		− 0.59	0.044
Parent-child relationship quality- Mother		0.23	0.254
Perceived differential affect - Father		− 0.42	0.054
Perceived differential affect - Mother		0.30	0.167

−2ΔLL refers to the difference in −2 log likelihood of the model presented and the unconditional model. Model 2 testing associations with family factors was conducted with 42 participants, since inter-parental violence was not measured in single parent families

The model testing family factors including interparental violence included only two-parent families (*N* = 42), therefore the model was also run without interparental violence (*N* = 52). The model without interparental violence showed equivalent results, therefore only the model with interparental violence is presented. The model with family factors showed that only the perceived quality of the father-child relationship was related to observed aggression, indicating that children who reported a higher quality of the relationship with their father showed less aggression towards their sibling in the VR experiment. However, given that the model did not show an improved fit compared to an empty model, we will interpret these results with caution.

## Discussion

The current study aimed to gain insight into the usability of an interactive VR experiment as a means of assessing young adolescents’ aggressive tendencies towards their sibling. We expected that such experiment could be of added value to assess sibling aggression in an ecologically valid manner, in accordance to a similar approach in assessing peer aggression in children (Verhoef et al., 2022). As this is a newly developed experiment, we conducted a first-phase pilot, in which we assessed the convergent and construct validity. Supporting convergent validity, results showed small to moderate associations between observed physical aggression and self-reported physical and psychological aggression towards the sibling. In addition, observed task-directed aggression was positively related to self-reported psychological aggression towards the sibling, with a small effect size. These results indicate that the aggressive behavior that was observed in virtual interactions with their assumed sibling, partly aligns with how adolescents describe their level of aggression towards their sibling. The imperfect convergence across these different instruments reinforces the call for a multimethod approach to assess sibling aggression in children.

Another result supporting the added value of this interactive VR experiment, is its indication of measurement sensitivity. Results showed relatively high frequencies of observed aggression and relatively large variances in all three aspects of aggression as measured by the VR experiment. Two short scenarios of three minutes maximum that included provocation by a virtual sibling, elicited several forms of aggressive responding. Most participants showed at least one form of physical aggression (such as hitting the virtual sibling), psychological aggression (including insulting their virtual sibling), or task-directed aggression (sabotaging the game of the virtual sibling). Moreover, although participants could only manipulate things in the VR by using the controllers, this did not stop them from showing other behaviors which were not visible in the VR. Participant showed natural behavior, such as sticking out their tongue, kicking, and pointing to their sibling. This can be seen as an indication that the participants forgot that these behaviors would not show an effect in the VR, that immersion was successful and that they responded as they would have when interacting with their sibling outside the virtual environment. The VR experiment thereby was successful in capturing reactive aggression and seemed sensitive to assess individual differences in aggressive responding. These findings are in line with the results in previous research (Verhoef et al., 2022), and highlight the potential of using VR experiments to assess aggression at clinical levels or in other contexts. This may be of interest for the juvenile justice system for example, in which assessment of aggression is part of risk assessment of recidivism in juvenile offenders (Viljoen & Vincent, 2024).

Contrary to expectations, parent- and sibling reported aggression were not significantly related to observed aggression, neither was vignette-based aggression. This may be explained by the different construct that was measured by parent-report, which assessed aggression in general instead of sibling aggression specifically. Furthermore, parents may underestimate the aggressive behavior between their children (Barhight et al., 2017) or may be less sensitive to detect psychological aggression between their children. Similarly, siblings may perceive aggressive behavior from their brother or sister as normative, and as a result may underreport this behavior, which may explain the lack of associations between aggression observed in the VR experiment and sibling reported aggression. With regard to vignette-based aggression the lack of associations may be explained by that vignettes ask how participants imagine they would respond, which is often not necessarily similar to actual behavior (Hughes & Huby, 2004). A second limitation of assessing aggression with vignettes, which may explain the lack of convergence with observed sibling aggression in the VR, is a lack of ecological validity of the social scenario presented in the video vignette. Another explanation for the limited convergence, may be that method variance plays a substantial role in the measurement of aggression (Barhight et al., 2017). These results advocate for a multimethod approach to assess aggression that not only includes questionnaires and vignettes, but also behavioral observations. Such an extensive approach may provide a more detailed insight into the complexity of human behavior.

A secondary goal of this study was to examine whether sibling aggression as measured in a VR experiment is related to individual, sibling, and family factors to provide insight in both the construct validity of the VR experiment and in the antecedents of sibling aggression. Results showed that individual factors (e.g., behavioral problems) and sibling factors (e.g., age difference between siblings and sibling gender combination) were not related to observed aggression. Of the different family factors only the perceived quality of the father-child relationship was negatively related to observed aggression, which is in line with the literature showing that positive parent-child relationships may be protective against sibling aggression (Portner & Riggs, 2016). The absence of associations with other family factors could be partially attributed to the relatively small sample size of this pilot study, which limits the ability to detect small effects. Due to the limited number of studies on sibling aggression and the inconclusive findings regarding associations with individual and sibling factors, drawing definitive conclusions about construct validity is challenging. Further research into antecedents of sibling aggression is recommended.

The study demonstrated several strengths, particularly in its successful manipulation of the VR experiment, which was notably effective among younger participants. A large majority of the younger participants believed that they were interacting with their sibling in VR, whereas a research assistant controlled the sibling-avatar. This manipulation increases the ecological validity of our experiment, the immersion of the participants, and level of experimental control, without enforcing real conflicts between siblings. In this case introducing a form of avatar-based deception was considered justified, and measures were taken to debrief the participants. Importantly, the study adhered strictly to ethical guidelines, ensuring that participants were safe while engaging in provocative social interactions. No participants were harmed, and there were no lingering negative feelings following the debriefing process, on the contrary most participants mentioned they enjoyed playing the competitive games in VR. This underscores the ethical rigor and participant welfare considerations integrated into the study design.

The study also had several limitations. The small sample size and the relatively large age-range of the participants limited the generalizability of the findings, as a larger sample could provide more robust data. Additionally, the non-clinical sample used in the study had generally low levels of aggression, raising questions about the applicability of the VR experiment in clinical populations. Moreover, the provocation in the experiment was mild, which may not be sensitive enough to capture more severe levels of sibling violence. Furthermore, interobserver reliability for two constructs of the observational coding system were moderate, possibly due to the small sample size and relatively low levels of observed aggression. Lastly, in the older participants, the credibility of the manipulation in the VR experiment was lower, potentially limiting a realistic response toward their virtual sibling. These factors highlight the need for further research with larger, more diverse samples and varying levels of provocation to better assess the VR experiment’s effectiveness in different contexts and populations. Furthermore, measures could be taken to enhance the credibility of the VR manipulation, such as making the avatar’s appearance more closely resemble that of the actual sibling.

In conclusion, this study offers valuable insights into the usability of an interactive VR experiment for assessing young adolescents’ aggressive tendencies towards their siblings. The findings underscore the potential of such VR experiments to capture reactive aggression in an ecologically valid manner. The experiment demonstrated sensitivity in measuring aggression, eliciting various forms of aggressive responses even within short, mildly provocative scenarios. Consequently, VR experiments could be valuable when implementing a multimethod approach for assessing aggression in other contexts as well, such as in juvenile justice populations (Lampe et al., 2023). However, the study’s limitations, including a small non-clinical sample and mild provocation levels, highlight the need for further research with more diverse and clinical populations to fully assess the VR experiment’s applicability and effectiveness. Moreover, it is important to recognize that incorporating technology into assessment presents both opportunities and challenges, including financial costs and implementation hurdles. Despite these limitations, the successful manipulation of the VR experiment provides a solid foundation for future investigations into the assessment of aggression using immersive virtual environments.

## Data Availability

Our data include sensitive data that could identify participants. Therefore, the data are not openly available in a public repository. However, data are available via the principal investigator (SRvB), upon reasonable request.

## References

[CR1] Achenbach, T. M. (2009). *The Achenbach system of empirically based assessment (ASEBA): Development, findings, theory, and applications*. University of Vermont, Research Center for Children, Youth, & Families.

[CR2] Alsem, S. C., Van Dijk, A., Verhulp, E. E., Dekkers, T. J., & De Castro, B. O. (2023). Treating children’s aggressive behavior problems using cognitive behavior therapy with virtual reality: A multicenter randomized controlled trial. *Child Development,**94*(6), e344–e361. 10.1111/cdev.13966 37459452 10.1111/cdev.13966

[CR3] Barhight, L. R., Hubbard, J. A., Swift, L. E., & Konold, T. R. (2017). A multitrait–multimethod approach to assessing childhood aggression and related constructs. *Merrill-Palmer Quarterly*, *63*(3), 367–395. 10.13110/merrpalmquar1982.63.3.0367

[CR4] Brett, H., Bartoli, A. J., & Smith, P. K. (2023). Sibling bullying during childhood: A scoping review. *Aggression and Violent Behavior,* Article 101862. 10.1016/j.avb.2023.101862

[CR5] Carvalho Relva, I., Fernandes, O. M., & Costa, R. (2013). Psychometric properties of revised conflict tactics scales: Portuguese sibling version (CTS2-SP). *Journal of Family Violence*, *28*, 577–585. 10.1007/s10896-013-9530-0

[CR6] Daniels, D., & Plomin, R. (1985). Differential experience of siblings in the same family. *Developmental Psychology*, *21*(5), 747–760. 10.1037/0012-1649.21.5.747

[CR7] Faul, F., Erdfelder, E., Buchner, A., & Lang, A. G. (2009). Statistical power analyses using G*Power 3.1: Tests for correlation and regression analyses. *Behavior Research Methods*, *41*, 1149–1160. 10.3758/BRM.41.4.1149 19897823 10.3758/BRM.41.4.1149

[CR8] Feinberg, M. E., McHale, S. M., Crouter, A. C., & Cumsille, P. (2003). Sibling differentiation: Sibling and parent relationship trajectories in adolescence. *Child development, 74*(5), 1261–1274. 10.1111/1467-8624.00606 10.1111/1467-8624.00606 14552397

[CR9] Fite, P. J., O’Dell, C., Doyle, R. L., & Tampke, E. C. (2021). Proactive and reactive aggression towards siblings versus peers. *Journal of Psychopathology and Behavioral Assessment*, *43*, 12–20. 10.1007/s10862-020-09849-w

[CR10] Granic, I. (2014). The role of anxiety in the development, maintenance, and treatment of childhood aggression. *Development and Psychopathology,**26*(4pt2), 1515–1530. 10.1017/S0954579414001175 25422976 10.1017/S0954579414001175

[CR11] Hehman, J. A., Burch, R. L., & Salmon, C. A. (2023). Sibling conflict and closeness: The effects of sex, number of siblings, relatedness, parental resemblance and investment. *Evolutionary Psychological Science,**9*(2), 224–235. 10.1007/s40806-022-00353-w

[CR12] Hughes, R., & Huby, M. (2004). The construction and interpretation of vignettes in social research. *Social Work and Social Sciences Review*, *11*(1), 36–51. 10.1921/swssr.v11i1.428

[CR13] Ingram, K. M., Espelage, D. L., Davis, J. P., & Merrin, G. J. (2020). Family violence, sibling, and peer aggression during adolescence: Associations with behavioral health outcomes. *Frontiers in Psychiatry*, *11*, 26. 10.3389/fpsyt.2020.00026 32116843 10.3389/fpsyt.2020.00026 PMC7027165

[CR14] Kempes, M. M., de Castro, B. O., & Sterck, E. H. (2008). Conflict management in 6–8-year-old aggressive Dutch boys: Do they reconcile? *Behaviour*, *1701–1722*. 10.1163/156853908786131306

[CR15] Kirsch, A. P., Kenrick, D. T., Ko, A., Pick, C. M., & Varnum, M. E. (2024). Sibling aggression is surprisingly common and sexually egalitarian. *Evolution and Human Behavior,**45*(2), 214–227. 10.1016/j.evolhumbehav.2024.03.001

[CR16] Lampe, K. G., Mulder, E. A., & Vermeiren, R. R. (2023). Ethnic differences in assessment: How self-report and observation converge and diverge among ethnically diverse incarcerated youths. *Journal of Threat Assessment and Management,**10*(2), 112–125. 10.1037/tam0000199

[CR17] Lange, A., Evers, A., Jansen, H., & Dolan, C. (2002). PACHIQ-R: The parent‐child interaction questionnaire—revised. *Family Process,**41*(4), 709–722. 10.1111/j.1545-5300.2002.00709.x 12613126 10.1111/j.1545-5300.2002.00709.x

[CR18] Lawrence, T. I., Hong, J. S., Espelage, D. L., & Voisin, D. R. (2023). Antecedents of sibling aggression and bullying victimization: The parallel and serial contributions of depressive symptoms and substance use. *Journal of Affective Disorders*, *333*, 193–201. 10.1016/j.jad.2023.04.023 37084977 10.1016/j.jad.2023.04.023

[CR19] Loeser, M. K., Whiteman, S. D., & McHale, S. M. (2016). Siblings’ perceptions of differential treatment, fairness, and adolescent adjustment: A moderated indirect effects model. *Journal of Child and Family Studies*, *25*, 2405–2414. 10.10007/s10826-016-0429-2 27867295 10.1007/s10826-016-0429-2 PMC5110249

[CR20] Perkins, N. H., & Grossman, S. F. (2020). Sibling violence: The missing piece in family violence policy. *Advances in Social Work*, *19*(1), 138–156. 10.18060/22611

[CR21] Perry, K. J., & Price, J. (2017). The role of placement history and current family environment in children’s aggression in foster care. *Journal of Child and Family Studies,**26*(4), 1135–1150. 10.1007/s10826-016-0642-z 29551877 10.1007/s10826-016-0642-z PMC5854210

[CR22] Perry, K. J., Ostrov, J. M., Murray-Close, D., Blakely-McClure, S. J., Kiefer, J., DeJesus-Rodriguez, A., & Wesolowski, A. (2021). Measurement of aggressive behavior in early childhood: A critical analysis using five informants. *Journal of Experimental Child Psychology*, *209*, 105180. 10.1016/j.jecp.2021.105180 34087603 10.1016/j.jecp.2021.105180

[CR23] Portner, L. C., & Riggs, S. A. (2016). Sibling relationships in emerging adulthood: Associations with parent–child relationship. *Journal of Child and Family Studies*, *25*(6), 1755–1764. 10.1007/s10826-015-0358-5

[CR24] Sanner, C., Russell, L. T., Coleman, M., & Ganong, L. (2018). Half-sibling and stepsibling relationships: A systematic integrative review. *Journal of Family Theory & Review,**10*(4), 765–784. 10.1111/jftr.12291

[CR25] Straus, M. A., Hamby, S. L., Boney-McCoy, S., & Sugarman, D. B. (1996). The revised conflict tactics scales (CTS2) development and preliminary psychometric data. *Journal of Family Issues*, *17*(3), 283–316. 10.1177/019251396017003001

[CR26] Tabachnick, B. G., Fidell, L. S., & Ullman, J. B. (2013). *Using multivariate statistics* (Vol. 6). pearson.

[CR27] Tanskanen, A. O., & Danielsbacka, M. (2021). Brothers and sisters across the life course: Eleven factors shaping relationship quality in adult siblings. In A. Buchanan & A. Rotkirch (Eds.), *Brothers and sisters: Sibling relationships across the life course* (pp. 25–40). Palgrave Macmillan. 10.1007/978-3-030-55985-4_2

[CR28] Tippett, N., & Wolke, D. (2015). Aggression between siblings: Associations with the home environment and peer bullying. *Aggressive Behavior*, *41*(1), 14–24. 10.1002/ab.21557 25187483 10.1002/ab.21557

[CR29] Tucker, C. J., Whitworth, T. R., & Finkelhor, D. (2025). Clarifying labels, constructs, and definitions: Sibling aggression and abuse are family violence. *Journal of Social and Personal Relationships*. 10.1177/02654075251332741

[CR30] Van Berkel, S. R., Tucker, C. J., & Finkelhor, D. (2018). The combination of sibling victimization and parental child maltreatment on mental health problems and delinquency. *Child Maltreatment,**23*(3), 244–253. 10.1177/1077559517751670 29310443 10.1177/1077559517751670 PMC6039865

[CR31] Van Rest, M. M., Vriens, A., Matthys, W., & Van Nieuwenhuijzen, M. (2020). The social information processing test SIVT provides insight into individual differences in social information processing by children and adolescents with behaviour problems. *Kind En Adolescent*, *41*, 122–140. 10.1007/s12453-019-00227-2

[CR32] Verhoef, R. E., Verhulp, E. E., van Dijk, A., & de Castro, B. O. (2022). Interactive virtual reality versus vignette-based assessment of children’s aggressive social information processing. *Research on Child and Adolescent Psychopathology*, *50*(5), 621–636. 10.1007/s10802-021-00879-w 34648102 10.1007/s10802-021-00879-w PMC9054903

[CR33] Verhulst, F. C., van der Ende, J., & Koot, H. M. (1996). *Handleiding voor de CBCL/4–18*. Sophia Kinderziekenhuis/Academisch Ziekenhuis/Erasmus Universiteit.

[CR34] Viljoen, J. L., & Vincent, G. M. (2024). Risk assessments for violence and reoffending: Implementation and impact on risk management. *Clinical Psychology: Science And Practice,**31*(2), 119–131. 10.1111/cpsp.12378

[CR35] Wolke, D., & Lereya, S. T. (2015). Long-term effects of bullying. *Archives of Disease in Childhood*, *100*(9), 879–885. 10.1136/archdischild-2014-306667 25670406 10.1136/archdischild-2014-306667 PMC4552909

